# Supplementary material for: Modeling glioblastoma heterogeneity as a dynamic network of cell states
Source: Mol Syst Biol. 2021 Sep 16;17(9):e10105. doi: 10.15252/msb.202010105 (PMC8444284; doi:10.15252/msb.202010105)
Supplement: Supplementary file 6 — Source Data for Figure 5 [file MSB-17-e10105-s004.zip › Figure5A_sourcedata/GSEA_3071/hallmarks_stateV.GseaPreranked.1623742546329/HALLMARK_UV_RESPONSE_UP.html]

Details for gene set HALLMARK\_UV\_RESPONSE\_UP[GSEA]

|  || Dataset | state43071 |
| Phenotype | NoPhenotypeAvailable |
| Upregulated in class | na\_pos |
| GeneSet | HALLMARK\_UV\_RESPONSE\_UP |
| Enrichment Score (ES) | 0.28182146 |
| Normalized Enrichment Score (NES) | 1.4055942 |
| Nominal p-value | 0.06806283 |
| FDR q-value | 0.08902729 |
| FWER p-Value | 0.855 |
Table: GSEA Results Summary

  

Fig 1: Enrichment plot: HALLMARK\_UV\_RESPONSE\_UP      
 Profile of the Running ES Score & Positions of GeneSet Members on the Rank Ordered List

  

| PROBE | GENE SYMBOL | GENE\_TITLE | RANK IN GENE LIST | RANK METRIC SCORE | RUNNING ES | CORE ENRICHMENT || 1 | IGFBP2 |  |  | 8 | 1.922 | 0.0451 | Yes |
| 2 | OLFM1 |  |  | 102 | 1.361 | 0.0604 | Yes |
| 3 | FEN1 |  |  | 139 | 1.296 | 0.0850 | Yes |
| 4 | RFC4 |  |  | 171 | 1.227 | 0.1089 | Yes |
| 5 | GGH |  |  | 182 | 1.207 | 0.1363 | Yes |
| 6 | BTG3 |  |  | 226 | 1.141 | 0.1557 | Yes |
| 7 | CDK2 |  |  | 233 | 1.134 | 0.1821 | Yes |
| 8 | CYP1A1 |  |  | 245 | 1.124 | 0.2073 | Yes |
| 9 | CCNE1 |  |  | 461 | 0.901 | 0.1881 | Yes |
| 10 | NPTXR |  |  | 528 | 0.849 | 0.1961 | Yes |
| 11 | SELENOW |  |  | 586 | 0.808 | 0.2048 | Yes |
| 12 | CHRNA5 |  |  | 595 | 0.804 | 0.2228 | Yes |
| 13 | SLC25A4 |  |  | 641 | 0.778 | 0.2331 | Yes |
| 14 | ENO2 |  |  | 728 | 0.731 | 0.2344 | Yes |
| 15 | AGO2 |  |  | 739 | 0.727 | 0.2501 | Yes |
| 16 | PARP2 |  |  | 864 | 0.673 | 0.2428 | Yes |
| 17 | KCNH2 |  |  | 955 | 0.638 | 0.2410 | Yes |
| 18 | ARRB2 |  |  | 994 | 0.622 | 0.2489 | Yes |
| 19 | CASP3 |  |  | 1057 | 0.605 | 0.2517 | Yes |
| 20 | HYAL2 |  |  | 1065 | 0.603 | 0.2650 | Yes |
| 21 | LHX2 |  |  | 1121 | 0.587 | 0.2688 | Yes |
| 22 | SHOX2 |  |  | 1128 | 0.586 | 0.2818 | Yes |
| 23 | TST |  |  | 1321 | 0.528 | 0.2580 | No |
| 24 | KLHDC3 |  |  | 1387 | 0.509 | 0.2579 | No |
| 25 | RPN1 |  |  | 1418 | 0.499 | 0.2643 | No |
| 26 | PRKACA |  |  | 1512 | 0.478 | 0.2581 | No |
| 27 | HNRNPU |  |  | 1548 | 0.470 | 0.2628 | No |
| 28 | CNP |  |  | 1879 | 0.406 | 0.2096 | No |
| 29 | NUP58 |  |  | 1892 | 0.405 | 0.2172 | No |
| 30 | DLG4 |  |  | 1966 | 0.394 | 0.2128 | No |
| 31 | CCND3 |  |  | 1991 | 0.390 | 0.2177 | No |
| 32 | CDC5L |  |  | 2115 | 0.372 | 0.2032 | No |
| 33 | HSPA13 |  |  | 2238 | 0.358 | 0.1886 | No |
| 34 | BSG |  |  | 2255 | 0.356 | 0.1941 | No |
| 35 | DGAT1 |  |  | 2278 | 0.353 | 0.1985 | No |
| 36 | CDC34 |  |  | 2426 | 0.332 | 0.1785 | No |
| 37 | PPIF |  |  | 2478 | 0.325 | 0.1766 | No |
| 38 | ACAA1 |  |  | 2502 | 0.322 | 0.1800 | No |
| 39 | FOS |  |  | 2529 | 0.319 | 0.1828 | No |
| 40 | BID |  |  | 2534 | 0.319 | 0.1898 | No |
| 41 | CTSV |  |  | 2541 | 0.317 | 0.1963 | No |
| 42 | PPP1R2 |  |  | 2550 | 0.316 | 0.2025 | No |
| 43 | DNAJB1 |  |  | 3143 | 0.256 | 0.0955 | No |
| 44 | HSPA2 |  |  | 3149 | 0.255 | 0.1008 | No |
| 45 | IL6ST |  |  | 3233 | -0.257 | 0.0912 | No |
| 46 | DNAJA1 |  |  | 3350 | -0.275 | 0.0757 | No |
| 47 | EIF5 |  |  | 3375 | -0.278 | 0.0778 | No |
| 48 | AMD1 |  |  | 3419 | -0.285 | 0.0765 | No |
| 49 | HMOX1 |  |  | 3426 | -0.286 | 0.0823 | No |
| 50 | GRINA |  |  | 3505 | -0.300 | 0.0747 | No |
| 51 | FURIN |  |  | 3861 | -0.361 | 0.0156 | No |
| 52 | UROD |  |  | 3921 | -0.371 | 0.0133 | No |
| 53 | JUNB |  |  | 4027 | -0.394 | 0.0029 | No |
| 54 | YKT6 |  |  | 4183 | -0.432 | -0.0163 | No |
| 55 | GRPEL1 |  |  | 4446 | -0.519 | -0.0538 | No |
| 56 | RHOB |  |  | 4820 | -0.731 | -0.1073 | No |
| 57 | CLTB |  |  | 4837 | -0.746 | -0.0923 | No |
| 58 | DDX21 |  |  | 4853 | -0.757 | -0.0767 | No |
| 59 | GAL |  |  | 4967 | -0.883 | -0.0769 | No |
| 60 | TUBA4A |  |  | 4987 | -0.909 | -0.0585 | No |
| 61 | SQSTM1 |  |  | 5099 | -1.149 | -0.0518 | No |
| 62 | IRF1 |  |  | 5100 | -1.155 | -0.0237 | No |
| 63 | CDKN2B |  |  | 5118 | -1.200 | 0.0021 | No |
| 64 | MMP14 |  |  | 5146 | -1.297 | 0.0285 | No |
Table: GSEA details [plain text format]

  

Fig 2: HALLMARK\_UV\_RESPONSE\_UP: Random ES distribution      
 Gene set null distribution of ES for **HALLMARK\_UV\_RESPONSE\_UP**
